# Bilingualism Affects Infant Cognition: Insights From New and Open Data

**DOI:** 10.1162/opmi_a_00057

**Published:** 2022-07-01

**Authors:** Rodrigo Dal Ben, Hilary Killam, Sadaf Pour Iliaei, Krista Byers-Heinlein

**Affiliations:** Concordia University

**Keywords:** infancy, bilingualism, cognitive control, inhibitory control, anticipatory looking

## Abstract

Bilingualism has been hypothesized to shape cognitive abilities across the lifespan. Here, we examined the replicability of a seminal study that showed monolingual–bilingual differences in infancy (Kovács & Mehler, 2009a) by collecting new data from 7-month-olds and 20-month-olds and reanalyzing three open datasets from 7- to 9-month-olds (D’Souza et al., 2020; Kalashnikova et al., 2020, 2021). Infants from all studies (*N* = 222) were tested in an anticipatory eye-tracking paradigm, where they learned to use a cue to anticipate a reward presented on one side of a screen during Training, and the opposite side at Test. To correctly anticipate the reward at Test, infants had to update their previously learned behavior. Across four out of five studies, a fine-grained analysis of infants’ anticipations showed that bilinguals were better able to update the previously learned response at Test, which could be related to bilinguals’ weaker initial learning during Training. However, in one study of 7-month-olds, we observed the opposite pattern: bilinguals performed better during Training, and monolinguals performed better at Test. These results show that bilingualism affects how infants process information during learning. We also highlight the potential of open science to advance our understanding of language development.

## INTRODUCTION

Bilingual infants are unique because they must acquire two languages simultaneously. Contrary to early warnings about potential disadvantages of growing up bilingual (Epstein, 1905; Macnamara, 1966; Yoshioka, 1929), there is evidence that bilingualism leads to improved metalinguistic awareness (Edwards & Christophersen, 1988; Yelland et al., 1993) as well as enhanced executive function and cognitive control (Bialystok, 2007; Bialystok & Barac, 2013). These adaptive impacts of bilingualism on cognitive function have been reported in studies with children (Barac & Bialystok, 2011; Bialystok, 2010; Bialystok & Martin, 2004; see reviews by Adesope et al., 2010; Barac et al., 2014), young and middle-aged adults (Costa et al., 2008), and older adults (Bialystok et al., 2007; Gold et al., 2013; Kavé et al., 2008). Moreover, studies have suggested that the age at which the two languages are acquired modulates this effect. For instance, bilingual adults who learned two languages in infancy show enhancements in cognitive control and brain connectivity relative to adults who learned their second language later in life (Kousaie et al., 2017).

However, other studies have raised concerns about the robustness of these findings, calling into question the existence of cognitive enhancements in bilinguals (e.g., de Bruin et al., 2015; Fernández-López & Perea, 2019; Leivada et al., 2020; Paap et al., 2015; Paap et al., 2018; Paap & Greenberg, 2013; Struys et al., 2018). Studies with infants have yielded mixed results, with some showing support for bilingualism’s impact on early cognition and others failing to do so (Brito & Barr, 2012; Brito et al., 2015; Brito et al., 2020; Comishen et al., 2019; D’Souza et al., 2020; Ibánez-Lillo et al., 2010; Kalashnikova et al., 2020; Kovács & Mehler, 2009a, 2009b; Molnar et al., 2014; Poulin-Dubois et al., 2011; Singh et al., 2014; Tsui & Fennell, 2019).

Thus, a careful examination of bilingualism’s effects on cognition, especially during infancy, is crucial for building comprehensive theories of linguistic and cognitive development. For example, demonstrating a monolingual–bilingual difference in preverbal infants would indicate that cognitive differences can be driven by factors related to receptive language, rather than solely by those related to productive language. Here we examined the replicability of a seminal study (Kovács & Mehler, 2009a) using new data from 7-month-olds and 20-month-olds as well as by reanalyzing three open datasets from 7- to 9-month-olds.

### Effects of Bilingualism on Cognition

Executive functions are mental processes that help individuals pay attention, flexibly ignore unnecessary information, and quickly adapt to changing circumstances (Diamond, 2013). One component of executive function—inhibitory control—has historically been the major focus of research on monolingual–bilingual cognitive differences (for reviews see Barac & Bialystok, 2011; Hilchey & Klein, 2011). According to early views, since bilinguals typically only speak one language at a time, they constantly need to select representations from their target language while inhibiting the other language, which in turn enhances domain-general inhibitory processes (Green, 1998; Philipp et al., 2007). However, more recent accounts propose that bilingual experience serves to strengthen other aspects of executive function as well and that a single cognitive selection mechanism may underpin the ability to use different languages in different contexts (Blanco-Elorrieta & Caramazza, 2021). This is supported by studies finding that bilinguals outperform monolinguals in congruent trials of conflict tasks that require inhibitory control, as well as in the incongruent trials that do not (Hilchey & Klein, 2011). According to Bialystok (2017), attention is at the core of executive function employed in these tasks, and bilingual experience provides the basis for the development of a more flexible system of attention as bilinguals recurrently need to switch their attention between two languages. Other theorists have conceptualized monolingual–bilingual differences in terms of neuroplasticity, while still emphasizing the impact of bilingualism on executive function (Baum & Titone, 2014). Understanding the developmental trajectory of monolingual–bilingual differences, especially during infancy, can shed light on these and other theories.

### Effects of Bilingualism on Infant Cognition

In a seminal study attempting to detect monolingual–bilingual cognitive differences at a much younger age than had been previously demonstrated, Kovács and Mehler (2009a) compared the performance of 7-month-old monolingual and bilingual infants in three eye-tracking experiments. Each experiment consisted of nine training and nine test trials assessing infants’ anticipatory eye movements to visual and speech cues. Training trials began with a visual or auditory cue, followed by a 1,000-ms anticipatory interval, and then a visual reward displayed consistently on one side of the screen (e.g., left). Infants were expected to learn that the cue predicted the reward and anticipate its appearance by looking toward the trained side during the anticipatory interval. Test trials had the same structure, except that the side of the reward was switched (e.g., right). To successfully anticipate the reward at test, infants had to update their previously learned response. Results revealed that in all experiments, monolinguals and bilinguals performed similarly during the training phase and learned to anticipate the reward. At Test, however, only bilinguals displayed an increase in correct anticipatory looks over the course of the nine trials. The authors concluded that perceiving and processing two languages from birth affects bilinguals’ cognitive control beyond the language domain, allowing bilinguals to suppress previously learned responses and update predictions on the experimental task. Contrary to some theoretical accounts (e.g., Green, 1998; Philipp et al., 2007), this finding indicates that monolingual–bilingual cognitive differences can be observed even prior to the onset of speech production, suggesting that bilingual infants’ experience processing and building separate mental representations of two languages is sufficient for enhancing executive function.

Given its theoretical importance, there have been several attempts to conceptually replicate this initial finding. Four teams of researchers, testing infants from 7 and 10 months of age in tasks similar (but not identical) to the original study, did not observe differences between monolinguals and bilinguals (D’Souza et al., 2020; Ibánez-Lillo et al. 2010; Molnar et al., 2014; Tsui & Fennell, 2019). However, a paper that included more training and test trials reported that bilinguals outperformed monolinguals in one of two studies, although only after post hoc exclusion of some trials (Comishen et al., 2019). A more recent investigation reported that at Test bilinguals performed either similarly to or better than monolinguals in one condition (depending on the analysis), but performed worse in another (see Kalashnikova et al., 2020; revised analysis 2021, https://onlinelibrary.wiley.com/doi/10.1111/desc.13139). There are multiple possibilities that could explain why these replication attempts have reported differing results, including sampling error in the context of small sample sizes, methodological differences across studies, differences between the bilingual populations tested, and different analytic choices. Overall, out of six teams that have attempted conceptual replications of Kovács and Mehler (2009a), only two have reported evidence supporting enhanced cognitive control in bilingual infants in this paradigm.

Other tasks have also been used to examine the impacts of bilingualism on infant cognition, and findings have been mixed. For example, in a visual habituation task, 6-month-old bilinguals showed more efficient visual stimulus encoding and stronger recognition of stimuli than monolinguals (Singh et al., 2014). In another series of studies, bilinguals tested between 7 and 9 months of age outperformed monolinguals in two of three cognitive flexibility tasks (D’Souza et al., 2020). Enhancements in memory generalization were found in 6- and 18-month-old bilinguals (Brito & Barr, 2012; Brito et al., 2015; Brito et al., 2020), but unexpectedly not in trilingual infants (Brito et al., 2015). In another study, 12-month-old bilinguals were able to associate different syllabic patterns (e.g., AAB as in *lo-lo-vu* vs. ABA as in *lo-vu-lo*) with rewards on different sides of the screen, whereas monolinguals were not (Kovács & Mehler, 2009b). Executive functions were found to be enhanced in bilinguals at 24 months old on a Shape Stroop task, although there were no monolingual–bilingual differences in any of four other executive function tasks tested (Poulin-Dubois et al., 2011). Moreover, while some of these studies used tasks designed to tap into executive functions (Kovács & Mehler, 2009b; Poulin-Dubois et al., 2011), others have found monolingual–bilingual differences in tasks that are not clearly related to executive functions (Brito & Barr, 2012; Brito et al., 2015; Brito et al., 2020; D’Souza et al., 2020). Such findings have motivated new theories to explain monolingual–bilingual cognitive differences, for example, that bilingual infants’ more variable language environments promote greater exploratory behavior (D’Souza et al., 2020).

Overall, evidence has been inconsistent regarding whether and how bilingualism impacts domain-general cognition in infancy. In addition, there have been recent concerns about reproducibility in psychological science (Open Science Collaboration, 2015; Simmons et al., 2011), bilingualism research (Bolibaugh et al., 2021; de Bruin et al., 2015; Fernández-López & Perea, 2019; Leivada et al., 2020), and infancy research (Frank et al., 2017). Given these concerns, replicating and extending previous work on this matter is crucial.

## CURRENT RESEARCH

The primary goal of the current study was to further investigate the existence and development of a bilingual–monolingual cognitive difference in infancy. We tested monolingual and bilingual infants in a simplified version of the paradigm used by Kovács and Mehler (2009a), using a single audiovisual cue for all infants. Whereas previous studies have tested infants at a single age, we tested both preverbal 7-month-olds (Study 1) and early verbal 20-month-olds (Study 3) to examine developmental effects. In an effort to increase statistical power and better characterize infants’ performance, we employed logistic mixed-effects regression to model performance at multiple time points per trial (DeBolt et al., 2020). This analytic approach has not previously been used to analyze data from this anticipation paradigm, and it allowed us to make fuller use of the fine-grained eye-tracking data to characterize infants’ moment-to-moment performance within and across trials. This was done as a complement to the analytic approach (ANOVAs [analysis of variance] on data averaged across three-trial blocks) used in the original Kovács and Mehler (2009a) study. As our data collection began prior to the publication of the replication attempts discussed in the previous section, we predicted that our results would replicate the original study: monolinguals and bilinguals would show similar performance during Training, but at Test bilinguals would outperform monolinguals when updating an anticipatory response.

Additionally, we conducted exploratory reanalyses (Studies 2a, 2b, and 2c) of open data from two recently published papers (D’Souza et al., 2020, Experiment 1; Kalashnikova et al., 2020, 2021, Visual Condition, Auditory Condition). Table 1 illustrates key aspects of participants and experimental design for each of these studies. Despite using almost identical experimental paradigms to Kovács and Mehler (2009a) and larger sample sizes, the authors reported conflicting results with samples of 7- to 9-month-old bilinguals and monolinguals. D’Souza et al. (2020) reported a failure to replicate the original findings, detecting no monolingual–bilingual differences at Test. Kalashnikova et al. (2020, 2021) reported results in their corrigendum for the Visual Condition which they interpreted as showing no monolingual–bilingual difference, although some analyses suggested that bilinguals may have outperformed monolinguals at Test. The authors found a different pattern in their Auditory Condition: monolinguals outperformed bilinguals at Test. Given the similarity of these studies to our Study 1, we applied the same fine-grained analytical approach (logistic mixed-effects models) to their data, aiming to capture additional effects that might have been undetected in their original analyses.

**Table 1.** Comparison of Datasets Used for Analyses

**Study**	**Original Authors**	**Age in months**	**Native languages**	**Unfiltered *N***	**Filtered *N***	**# Trials/phase**	**Example Cue**	**Results: Training phase**	**Results: Test phase**
1	Dal Ben et al. (2022; this article)	7	M: Fr or En; B: Fr–En or Fr/En–other	72 (36M, 36B)	43 (21M, 22B)	9	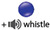	M > B	B > M
2a	D’Souza et al. (2020; Study 1)	7–9	M: En; B: En–other	102 (51M, 51B)	53 (29M, 24B)	9	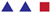	M > B	B > M
2b	Kalashnikova et al. (2020; Visual Condition)	7	M: Sp or Bq; B: Sp–Bq	70 (40M, 30B)	41 (25M, 16B)	12	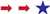	M > B	B > M
2c	Kalashnikova et al. (2020; Auditory Condition)	7	M: Sp or Bq; B: Sp–Bq	67 (38M, 29B)	44 (25M, 19B)	12	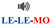	B > M	M > B
3	Dal Ben et al. (2022; this article)	20	M: Fr or En; B: Fr–En	72 (34M, 38B)	41 (22M, 19B)	9	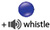	M = B	B > M

*Note*. M denotes monolinguals; B denotes bilinguals. En denotes English; Fr denotes French; Sp denotes Spanish, Bq denotes Basque; = and > denote the relative performance of monolinguals and bilinguals.

## STUDY 1: 7-MONTH-OLDS, NEW DATA

### Method

This research was conducted according to the Declaration of Helsinki, and was approved by the Human Research Ethics Board of Concordia University, Montreal (certificates UH2011-041, 10000439). Parents gave informed consent prior to participation. Stimuli, data, and analysis scripts are available at https://osf.io/bz8jn. Data were collected between January 2017 and July 2018.

#### Participants.

A total of 108 7-month-old infants were tested. We filtered out 29 infants from the main analysis because they failed to provide sufficient data, defined as gazing at the areas of interest for at least 50% of the analyzed anticipation period, on at least five of the nine trials for both the Training and the Test phases. This inclusion criterion was set prior to data collection to ensure that infants were paying attention, and was based on our experience testing infants in a variety of tasks. An additional 36 infants were excluded due to fussiness (*n* = 6), health issues (*n* = 5), failure to meet language criteria for being classified as monolingual or bilingual (*n* = 18; see next paragraph for exact criteria), technical issues with the eye-tracker (*n* = 6), and being older than the specified age range (*n =* 1).

The final sample consisted of 43 infants (*M*_age_ = 7m 11d, range: 6m 21d–8m 6d, 23 girls). Twenty-one infants were monolingual (*M*_age_ = 7m 10d, range: 6m 23d–8m 1d, 8 girls), classified as hearing either French (*n* = 12) or English (*n* = 9) at least 90% of the time from birth. The other 22 infants were bilingual (*M*_age_ = 7m 11d, range: 6m 21d–8m 6d, 15 girls), classified as hearing each of two languages from 25% and 75% of the time from birth (Pearson et al., 1993). Twelve bilinguals (55%) were learning English and French, and the other 10 were learning different language pairs (see the Supplemental Materials for language backgrounds). Participants lived in Montreal, Canada, and were recruited from government birth lists, community events, and social media. They were all healthy full-term infants (at least 37 weeks’ gestation) with no reported developmental, vision, or hearing impairments. Monolingual and bilingual samples were predominantly of mid- to high-socioeconomic status (as estimated via maternal educational attainment) and were comparable to each other: mothers of monolinguals had an average of 15.8 years of education and mothers of bilinguals had an average of 16.3 years. There was no significant difference in estimated years of maternal education across the two groups, *t*(40) = .72, *p* = .475, *d* = 0.11. Datasets and scripts are available at https://osf.io/bz8jn.

#### Measures.

To evaluate infants’ exposure to different languages, we used the Language Exposure Questionnaire (LEQ; Bosch & Sebastián-Gallés, 2001) in conjunction with the Multilingual Approach to Parent Language Estimates (MAPLE; Byers-Heinlein et al., 2019). The LEQ is a semistructured interview that asks parents about their family language background and the languages spoken directly to the child over the course of typical weekdays and weekends, both at home and in other environments such as daycare. This allows the calculation of the percentage of time infants are exposed to each language from birth. MAPLE provides guidelines for eliciting reliable information from families in a culturally sensitive manner.

#### Stimuli and Apparatus.

Stimuli were developed based on those used by Kovács and Mehler (2009a) and Reuter et al. (2018), with the goal of creating a simple and compelling nonlinguistic task (all stimuli are available at https://osf.io/bz8jn). In their Visual (nonlinguistic) Condition, Kovács and Mehler presented infants with a series of shapes following an AAB or ABB pattern, with the specific shapes changing on different trials. Reuter et al. were able to elicit similar anticipatory behavior by cuing infants with only a looming circle accompanied by a whistle sound, which was presented on all trials. Since we aimed for a conceptual rather than an exact replication of Kovács and Mehler, and there was no theoretical reason why a particular cue structure would be necessary to observe monolingual–bilingual differences, we chose to use a simple and consistent cue that might elicit more robust anticipations. Thus, our stimuli were similar to those used by Reuter et al.

**Figure 1.**
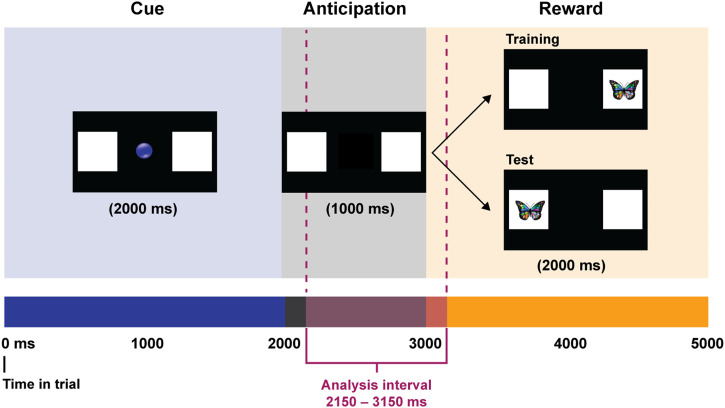
**Structure of the trial sequence.** Trials started with a display of a central visual fixation cue (a blue circle) against a black background and flanked by two white squares on the right and left sides of the screen. The anticipatory period began after the offset of the cue, where only the white squares were visible. At the end of the anticipatory period, a visual reward (a butterfly) appeared inside either the left or the right square. It was always displayed inside the same square during the Training phase (9 trials) and in the other square during the Test phase (9 trials). The analyzed anticipation interval is offset from the stimulus presentation by 150 ms, to account for the time it takes infants to initiate a gaze change.

Our cue consisted of a looming blue circle accompanied by a whistle sound, which was displayed for the first 2,000 ms of each trial. It was presented centrally on a black background and flanked by two white squares. After the offset of the cue, a 1,000-ms anticipatory period began, in which infants saw only the two squares. Finally, an audiovisual reward (a spinning butterfly accompanied by a tinkling sound) appeared inside either the left or the right square for 2,000 ms. Figure 1 depicts the structure of the trial sequence. During the Training trials, the reward appeared consistently on one side of the screen (e.g., in the left square), and during the Test trials it switched sides (e.g., in the right square). The side where the reward appeared was counterbalanced across participants, so that infants were randomly assigned to one of two experimental orders (e.g., right during Training and left during Test, or left during Training and right during Test). Trials were presented on a 24″ Tobii T60XL eye-tracker and eye gaze data were collected at 60 Hz using Tobii Studio Software.

#### Procedure.

During the study, infants sat on their parent’s lap on a chair in a sound-attenuated room, approximately 60 cm away from the eye-tracker. Parents wore darkened sunglasses and headphones playing masking music, and were instructed not to talk to their child during the study. The experiment started with the eye-tracker calibration, using a 5-point infant calibration routine. Next, infants completed 9 Training and 9 Test trials. The total duration of the experiment was approximately 90 s. Parents completed questionnaires either prior to or following the eye-tracking experiment. At the end of the session, parents were thanked for their participation and children received a small gift and an honorary diploma.

#### Data Analysis.

The main dependent variable was infants’ anticipatory eye movements. We defined anticipatory eye movements as looks to either of the white squares where the reward could appear during the 1,000-ms time window between the cue and the visual reward (following Kovács & Mehler, 2009a; McMurray & Aslin, 2004). Figure 1 shows the trial sequence and highlights the anticipation period. The anticipation period occurred between 2,000 ms and 3,000 ms after trial onset, immediately following the offset of the audio-visual cue, and immediately prior to the onset of the visual reward. Following Kovács & Mehler (2009a), the analyzed anticipation window was shifted to begin 150 ms after the offset of the cue and to end 150 ms after the onset of the reward, to account for the time necessary for infants to initiate an eye movement (Canfield et al., 1997). Thus, our analyzed anticipatory period was 2,150–3,150 ms after trial onset.

Infants’ looking was measured within three areas of interest (AOIs), corresponding to the squares on each side of the screen and the central fixation area where the blue circle appeared, using a square approximately 2 cm larger than the visual stimuli. As mentioned in the Participants section, prior to conducting the study, we planned both trial-level and infant-level inclusion criteria to ensure that only infants who were on task were retained for analysis. Although Kovács and Mehler (2009a) did not specifically mention applying such criteria, this is a common, although variable, approach in infant research (D’Souza et al., 2020; Kalashnikova et al., 2020), as infants who are not engaged in the task are unlikely to provide informative data (see Byers-Heinlein et al., 2020, and ManyBabies Consortium, 2020, for a discussion of missing data in infant looking time paradigms). Given our interest in how anticipatory behavior unfolds over time, we planned, prior to data collection, to limit analyses to trials with at least 500 ms total looking time to any of the AOIs (left, center, right) during the 1-s analyzed anticipatory period. In addition, infants who contributed fewer than five out of nine trials for both Training and Test phases were also excluded. To better understand the effects of this strict inclusion criteria, we also conducted parallel analyses using an unfiltered dataset, which did not impose any trial-level or infant-level exclusion criteria. Overall, we found a similar pattern of results, although in some cases significance patterns changed. Further discussion of the effect of data filtering, full models, and unfiltered data are available in the Supplemental Materials.

We implemented two analytical approaches (datasets and scripts are available at https://osf.io/bz8jn). The first used ANOVAs to analyze data averaged across blocks, following Kovács and Mehler (2009a) as closely as possible. The second approach used logistic mixed-effects models to harness the rich eye-tracking data (models were fit using the lme4 package for R; Bates et al., 2015).

#### Overview of ANOVA Approach.

Each trial was coded as correct (when infants looked longer to the white square where the reward would appear than to the other square) or incorrect (when infants looked longer to the square where the reward would not appear, or did not look at either square but looked only toward the central fixation AOI). Trials were grouped into three blocks (first/middle/last) in each of the two phases. This approach analyzed a maximum of 3 blocks/phase * 2 phases = 6 data points per infant.

A correct anticipation score was calculated for each block by dividing the number of trials with correct anticipations by the total number of valid trials. Note that in this context 50% cannot be considered “chance,” as correct performance involves comparing looks to the target side to looks at both the distractor side and the central fixation area combined. As such, infants responding randomly would be expected to have “incorrect” looks on more than 50% of trials (for example, if they perseverate on the central fixation area). ANOVA tables are presented in the Supplemental Materials, and for brevity this information is not repeated in-text.

#### Overview of Logistic Mixed-Effects Regression Approach.

We used logistic mixed-effects models to account for fixed and random effects arising from the repeated measures study design (e.g., Barr, 2008; Dixon, 2008; Humphrey & Swingley, 2018). Our analysis allowed us to investigate how anticipatory behavior unfolded within each learning instance (*within trials*) and how it accumulated across the experiment (*across trials*)—a major improvement over block-level analyses. In this approach, rather than categorizing each trial as correct or incorrect overall, we calculated the weighted proportion of looks to the correct side at each of five 200-ms time bins per trial.^1^ We omitted the first trial of each phase, since at that point infants did not have information with which to make a correct anticipation. This approach analyzed up to 5 time bins/trial * 8 trials/phase * 2 phases = 80 data points per infant. As such, it provided a richer characterization of infants’ performance over time and greater statistical power (DeBolt et al., 2020).

Our models tested the effects of language group, time bin, trial number, and their interactions on the proportion of looking to the correct side during each phase (Training and Test) separately. We aimed for a consistent random effects structure across models to facilitate comparisons. Our final models included only the random intercept for participants, as more complex models that included random slopes for trial number and time bin did not converge for all models. As mentioned, the first trial from both phases was excluded prior to fitting the models. Trial number was then scaled so that the reference trial would be the first analyzed trial (trial number − 2) and time bin was centered so that the middle time bin would be the reference time bin, as by then many infants might have made an anticipatory look (time bin − 2). Monolinguals were the reference language group. Thus, the model intercept describes the odds of making a correct anticipation for monolinguals at the middle time bin of Trial 2.

We report our results in odds ratios. Changes in the *relative* odds of making a correct anticipation as a function of language group, time bin, and trial number indicate how learning unfolded across the experiment. Specifically, the effect of time bin indexes infants’ anticipations *within* trials. The effect of trial number indexes learning *across* trials. The interaction between time bin and trial number indexes the *speed* of making correct anticipations across trials. Importantly, our models also included main effects and interactions of these terms with language group to capture different patterns of performance between monolinguals and bilinguals. For brevity, estimates and statistics for all studies are presented in Table 2 (Training) and Table 3 (Test), and are not repeated in the text. Model visualizations are presented in the Supplemental Materials.

**Table 2.** Fixed Effects as Odds Ratios From the Final Model [looking proportion ∼ language group * time bin * trial number + (1 | participant)] for the Training Phase of All Studies

Predictors	Interpretation	**Study 1**	**Study 2a**	**Study 2b**	**Study 2c**	**Study 3**
		Odds Ratios [CI] *p*	Odds Ratios [CI] *p*	Odds Ratios [CI] *p*	Odds Ratios [CI] *p*	Odds Ratios [CI] *p*
(Intercept)	The reference odds of *monolinguals* correctly anticipating during the *middle time bin* during *Trial 2*	0.08 [0.02–0.25]***	0.09 [0.04–0.19]***	0.06 [0.03–0.13]***	0.23 [0.14–0.38]***	1.09 [0.65–1.82] ns
language [bilingual]	The relative odds (compared to monolinguals) of *bilinguals* correctly anticipating during the *middle time bin* during *Trial 2*	0.39 [0.07–2.02] ns	0.73 [0.25–2.18] ns	0.61 [0.19–2.01] ns	0.59 [0.27–1.28] ns	0.84 [0.40–1.80] ns
time bin	The change in odds of *monolinguals* correctly anticipating during *Trial 2* for each unit change in *time bin*	6.47 [5.67–7.40]***	2.06 [1.94–2.19]***	2.67 [2.52–2.83]***	1.43 [1.37–1.50]***	3.15 [2.88–3.44]***
trial number	The change in odds of *monolinguals* correctly anticipating during the *middle time bin* for each unit change in *trial number*	1.12 [1.08–1.16]***	1.14 [1.12–1.16]***	1.16 [1.15–1.18]***	1.05 [1.04–1.06]***	0.88 [0.86–0.91]***
language [bilingual] * time bin	The relative change in odds of *bilinguals* correctly anticipating during *Trial 2* with each unit change in *time bin*	0.43 [0.36–0.51]***	0.91 [0.83–0.99]***	0.83 [0.75–0.92]***	1.51 [1.40–1.63]***	0.83 [0.74–0.94]**
language [bilingual] * trial number	The relative change in odds of *bilinguals* correctly anticipating during the *middle time bin* with each unit change in *trial number*	1.04 [0.99–1.10] ns	1.14 [1.11–1.17]***	0.96 [0.94–0.98]***	1.19 [1.17–1.21]***	1.07 [1.03–1.11]**
time bin * trial number	The change in odds of *monolinguals* correctly anticipating with each unit change in both *time bin* and *trial number* together (*difference in odds between earlier time bins in earlier trials and later time bins in later trials)*	0.91 [0.89–0.94]***	0.96 [0.95–0.97]***	0.94 [0.93–0.95]***	1.06 [1.05–1.07]***	1.01 [0.99–1.03] ns
language [bilingual] * time bin * trial number	The relative change in odds of *bilinguals* correctly anticipating with each unit change in both *time bin* and *trial number* together	1.11 [1.07–1.15]***	1.01 [1.00–1.03] ns	1.04 [1.02–1.05]***	0.92 [0.91–0.93]***	0.97 [0.94–1.00] ns
**Random Effects**						
*N*		43_id_	53_id_	41_id_	44_id_	41_id_
Observations		1,436	1,734	1,908	2,122	1,370
Marginal *R*^2^ / Conditional *R*^2^		0.267 / 0.772	0.106 / 0.594	0.160 / 0.593	0.178 / 0.456	0.325 / 0.532

*Note*. Tables 1 and 2 present the odds ratio estimates from logistic mixed-effects models. Odds ratios greater than 1 mean an outcome is more likely to happen; odds ratios less than 1 mean an outcome is less likely to happen; odds ratios of exactly 1 mean an outcome is as likely to happen as not (chance). Odds ratios are exponentiated from the logit, which renders effects that would be *additive* in the direct model output *multiplicative* in these tables. When interpreting the effects for interaction terms in the tables, the displayed odds ratio must be multiplied with the reference odds (or reference change in odds) to arrive at a final effect size.

ns = not significant, *p* > .05. *p ≤ .05. **p ≤ .01. ***p ≤ .001.

**Table 3.** Fixed Effects as Odds Ratios From the Final Model [looking proportion ∼ language group * time bin * trial number + (1 | participant)] for the Test Phase of All Studies

Predictors	Interpretation	**Study 1**	**Study 2a**	**Study 2b**	**Study 2c**	**Study 3**
		Odds Ratios [CI] *p*	Odds Ratios [CI] *p*	Odds Ratios [CI] *p*	Odds Ratios [CI] *p*	Odds Ratios [CI] *p*
[Intercept]	The reference odds of *monolinguals* correctly anticipating during the *middle time bin* during *Trial 2*	0.01 [0.00–0.03]***	0.04 [0.02–0.11]***	0.06 [0.04–0.11]***	0.19 [0.10–0.34]***	0.49 [0.32–0.76]**
language [bilingual]	The relative odds [compared to monolinguals] of *bilinguals* correctly anticipating during the *middle time bin* during *Trial 2*	2.02 [0.23–17.42] ns	1.59 [0.43–5.86] ns	0.74 [0.31–1.78] ns	0.31 [0.12–0.78]*	2.53 [1.33–4.81]**
time bin	The change in odds of *monolinguals* correctly anticipating during *Trial 2* for each unit change in *time bin*	1.26 [1.11–1.42]***	0.81 [0.77–0.86]***	1.65 [1.55–1.75]***	2.47 [2.34–2.60]***	1.67 [1.54–1.80]***
trial number	The change in odds of *monolinguals* correctly anticipating during the *middle time bin* for each unit change in *trial number*	1.05 [1.00–1.10]*	0.94 [0.92–0.96]***	1.12 [1.10–1.13]***	1.09 [1.08–1.11]***	0.98 [0.95–1.01] ns
language [bilingual] * time bin	The relative change in odds of *bilinguals* correctly anticipating during *Trial 2* with each unit change in *time bin*	2.47 [2.05–2.98]***	1.93 [1.78–2.09]***	1.28 [1.15–1.43]***	0.61 [0.56–0.67]***	1.09 [0.98–1.22] ns
language [bilingual] * trial number	The relative change in odds of *bilinguals* correctly anticipating during the *middle time bin* with each unit change in *trial number*	1.12 [1.05–1.19]***	1.32 [1.28–1.36]***	1.14 [1.11–1.17]***	1.07 [1.05–1.09]***	0.88 [0.85–0.92]***
time bin * trial number	The change in odds of *monolinguals* correctly anticipating with each unit change in both *time bin* and *trial number* together *[difference in odds between earlier time bins in earlier trials and later time bins in later trials]*	1.05 [1.02–1.09]***	1.12 [1.11–1.14]***	0.99 [0.98–1.00]**	0.95 [0.94–0.95]***	1.04 [1.02–1.06]***
[language [bilingual] * time bin] * trial number	The relative change in odds of *bilinguals* correctly anticipating with each unit change in both *time bin* and *trial number* together	0.95 [0.91–1.00]*	0.90 [0.89–0.92]***	0.99 [0.98–1.01] ns	1.09 [1.07–1.10]***	1.01 [0.98–1.04] ns
**Random Effects**						
*N*		43_id_	53_id_	41_id_	44_id_	41_id_
Observations		1383	1676	1672	1846	1272
Marginal *R*^2^ / Conditional *R*^2^		0.108 / 0.807	0.095 / 0.666	0.139 / 0.451	0.161 / 0.510	0.202 / 0.393

*Note*. ns = not significant, *p* > .05. *p ≤ .05. **p ≤ .01. ***p ≤ .001.

### Results

#### ANOVA.

**Figure 2.**
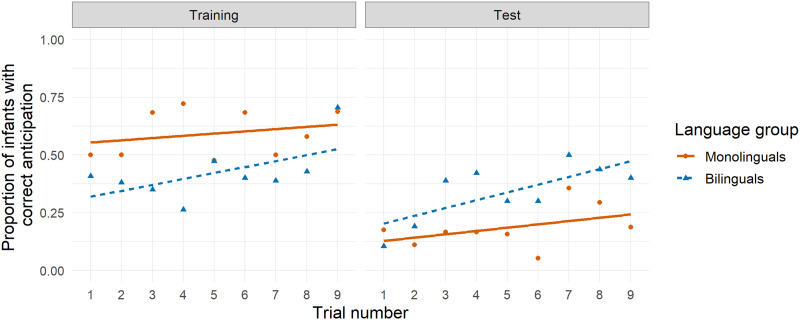
**Correct anticipation by trial for Study 1 (age 7 months).** Visualization of Study 1 (7 months) under Analytic Approach 1 (ANOVAs), similar to Figure 2 from Kovács and Mehler (2009a). Symbols represent the proportion of infants with correct anticipatory looks, with monolinguals plotted in red (circles and solid lines) and bilinguals plotted in blue (triangles and dashed lines). Lines show best linear fit for each group.

One participant was excluded from the ANOVAs due to missing data. During Training, a 2 (language group) × 3 (block) mixed ANOVA showed no significant interaction, nor significant main effects of language or block (Figure 2; ANOVA tables available in the Supplemental Materials). The latter was surprising because performance should improve from the first to the last block as learning unfolds. At Test, there was a significant main effect of block, demonstrating improved performance over the course of the Test phase, but no main effect of language group or interaction of block with language group. Overall, under the ANOVA approach we did not detect any monolingual–bilingual differences, and thus found no support for our prediction that bilinguals would outperform monolinguals at Test.

#### Logistic Mixed-Effects Regression.

**Figure 3.**
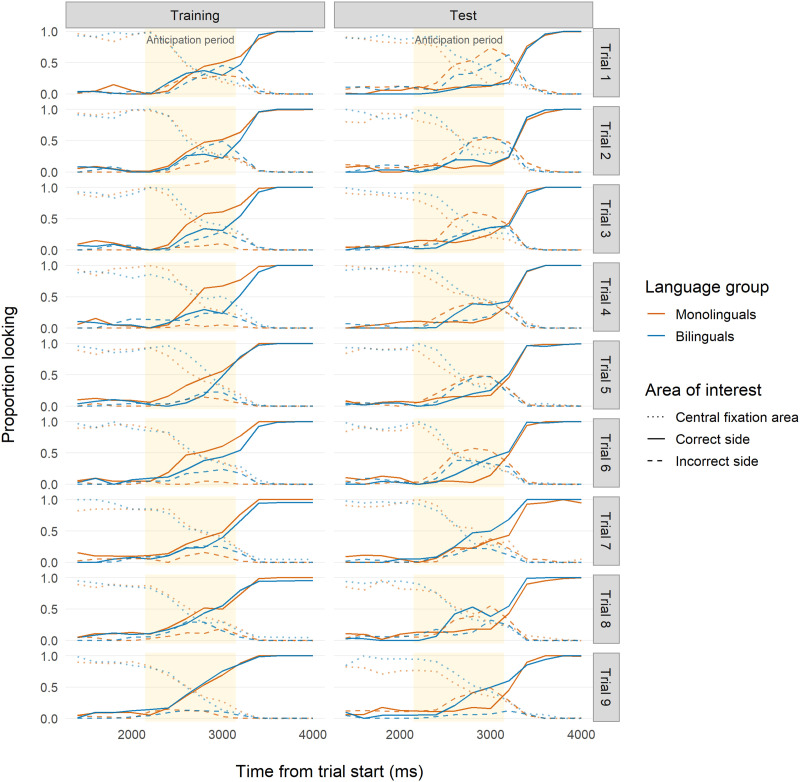
**Time course of infant looking for Study 1 (age 7 months).** Time course of proportion looking for Study 1 (7 months). Red indicates monolingual infants and blue indicates bilingual infants. Solid lines indicate looks to the correct side. Dashed lines indicate looks to the incorrect side. Dotted lines indicate looks to the location where the central fixation cue had appeared. Yellow backgrounds indicate the analyzed anticipation period time frame (2,150–3,150 ms after trial onset) used for our analyses.

During Training (Table 2; Figure 3), we unexpectedly found that 7-month-old monolinguals outperformed bilinguals, evidenced by more robust anticipations both within trials and across trials. Both groups showed evidence of learning across trials, and interaction effects indicated that bilinguals approached similar levels of performance as monolinguals by the end of Training (Trial 9). Conversely, at Test (Table 3; Figure 3), bilinguals showed much stronger evidence of correct anticipations, increasing their looking to the target more than monolinguals both within trials and across trials, with a sharper increase in later trials. This indicated that bilinguals were better at updating their previously learned association.

As predicted, our main analyses showed that 7-month-old bilinguals were better than monolinguals at updating their old behavior with a new one. Unexpectedly, our results suggest that these monolingual–bilingual differences might be due to different learning trajectories during the Training phase. Monolinguals initially learned to anticipate the reward faster than bilinguals (although bilinguals caught up by Trial 9), but struggled to update their behavior when the environment changed at Test. The reverse pattern was found for bilinguals. Crucially, previous studies have only found a difference at Test (e.g., Kalashnikova et al., 2020, 2021; Kovács & Mehler, 2009a). Thus, our finding that performance also differs during training suggests that the impacts of bilingualism on domain-general cognition in infancy are more complex than previously observed, in that it might involve both the ability to initially learn new information and to update existing knowledge.

## STUDY 2: 7- TO 9-MONTH-OLDS, REANALYZED DATA

Recently, close replications of Kovács and Mehler (2009a) were published by D’Souza et al. (2020) and Kalashnikova et al. (2020, 2021). Both studies tested whether 7- to 9-month-old bilinguals would be better at inhibiting a learned anticipation compared to matched monolinguals. D’Souza et al., despite using a near-identical task, a more sensitive trial-level analysis, and a larger sample than the original study, found no differences between groups’ anticipatory looks. In a corrected analysis (2021) of their original data (2020), Kalashnikova et al., on the other hand, reported that Basque–Spanish bilinguals performed similarly to monolinguals when tested using visual cues, although some analyses suggested that bilinguals showed better performance at Test. Depending on interpretation, this could be seen as replicating Kovács and Mehler’s original findings. However, when testing the same infants using auditory cues (using a within-subjects design), Kalashnikova et al. found that monolinguals outperformed bilinguals during the Test phase, thus failing to replicate the original study.

Given the close similarities in experimental design between our Study 1, D’Souza et al.’s (2020) Experiment 1, and Kalashnikova et al.’s (2020) Visual and Auditory Conditions, we conducted a reanalysis of their data using the same logistic mixed-effects regression we used with our own data, to see if there were additional effects that might have been undetected in their original analyses. This was possible thanks to the authors’ engagement with open science practices—their data were openly available and they were responsive when contacted for additional information.

It is worth noting that although all three of these studies were similar in design to Kovács and Mehler (2009a), the authors used different inclusion criteria for infant attention. D’Souza et al. (2020) excluded infants who did not have valid data on at least 75% of trials, while Kalashnikova et al. (2020, 2021) excluded infants who had more than 40% gaze loss for the entire task. To maximize comparability between results, we applied our stricter filtering criteria (i.e., at least 50% looking during the anticipation period in more than half of trials in both phases) to their data before reanalyzing it. This filtering resulted in smaller sample sizes than reported in the original experiments. To ensure that our results were not due to our stricter filtering and differences in sample size, we also ran the models on the unfiltered datasets. A comparison between the filtered and unfiltered samples is available in the Supplemental Materials, and an overall summary appears in Table 4.

**Table 4.** Differences Between Filtered and Unfiltered Samples in Our Analyses

**Study**	***n* (Unfiltered)**	***n* (Filtered)**	**Sample reduction**	**Analysis**	**Phase**	**Difference**	**Details**
1	72 (36M, 36B)	43 (21M, 22B)	40%	ANOVA	Training	**yes**	The unfiltered sample, but not the filtered sample, showed a group difference with monolinguals outperforming bilinguals.
					Test	no	
				LMEM	Training	no	
					Test	no	
2a	102 (51M, 51B)	53 (29M, 24B)	48%	LMEM	Training	no	
					Test	no	
2b	70 (40M, 30B)	41 (25M, 16B)	41%	LMEM	Training	no	
					Test	**yes**	In the unfiltered sample only, the odds of bilinguals correctly anticipating increased less over the course of the baseline trial than the odds of monolinguals, but this difference was mediated to some extent by a significant three-way bilingual–trial number–time bin interaction. Thus, the odds of bilinguals correctly anticipating in later time bins increased as trial number increased from the baseline trial.
2c	67 (38M, 29B)	44 (25M, 19B)	34%	LMEM	Training	no	
					Test	no	
3	72 (34M, 38B)	41 (22M, 19B)	43%	ANOVA	Training	no	
					Test	**yes**	At Test, the effect of language group was no longer significant in the unfiltered sample.
				LMEM	Training	**yes**	In the unfiltered sample, the trial number–time bin interaction changed from nonsignificant to decreasing odds for monolinguals.
					Test	**yes**	Results from the unfiltered dataset were somewhat different: the only significant effect related to language group was its three-way interaction with trial number and time bin.

*Note*. M denotes monolinguals; B denotes bilinguals. LMEM = logistic mixed-effects model; ANOVA = analysis of variance.

## STUDY 2A: D’SOUZA ET AL. (2020) REANALYSIS

D’Souza et al.’s (2020) experimental design was very similar to our Study 1. Training and Test phases had 9 trials each. Following Kovács and Mehler (2009a, Experiment 3), trials started with an attention-getter stimulus (displayed for 500 ms), followed by a sequence of silent AAB or ABB visual cues (3,000 ms), an anticipation period (1,000 ms), and a reward (left or right side; 2,000 ms). Bilingual (*n* = 51) and monolingual (*n* = 51) infants were closely matched on age, gender, and parental socioeconomic status, and were counterbalanced across visual cue sequences and reward sides. To reanalyze D’Souza et al. (2020), we applied the inclusion criteria for attentiveness used in Study 1, which left 29 monolinguals and 24 bilinguals from the original sample. Both groups were still comparable in age, gender, and parental socioeconomic status.

**Figure 4.**
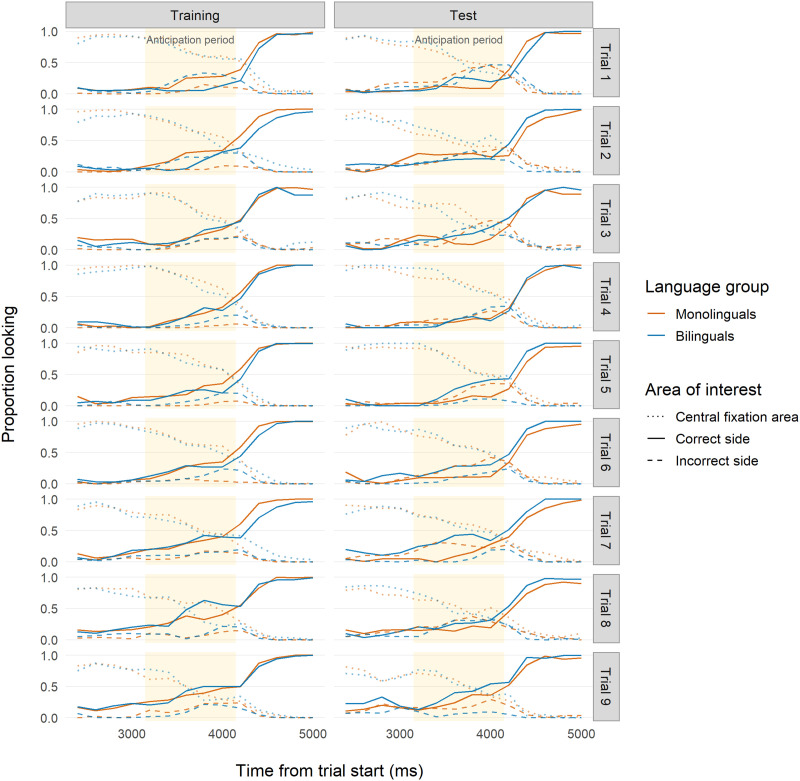
**Time course of infant looking for Study 2a (age 7–9 months).** Time course of proportion looking for Study 2a (reanalysis of D’Souza et al., 2020). Red indicates monolingual infants and blue indicates bilingual infants. Solid lines indicate looks to the correct side. Dashed lines indicate looks to the incorrect side. Dotted lines indicate looks to the location where the central fixation cue had appeared. Yellow backgrounds indicate the analyzed anticipation period time frame (3,150–4,150 ms after trial onset) used for our analyses.

Using our logistic mixed-effects model approach, we found a very similar pattern to Study 1 (Figure 4). During Training, monolinguals outperformed bilinguals (Table 2, see details in the Supplemental Materials). Specifically, bilinguals struggled more than monolinguals with correctly anticipating during initial Training trials, but their performance did catch up to monolinguals by Trial 9, a finding also reported by D’Souza et al. (2020) in their original analysis. In the Test phase, bilinguals outperformed monolinguals across both time bin and trial number (Table 3). Significant interactions indicated that only bilinguals showed improvement over the course of the trials, while monolinguals’ performance actually decreased, suggesting that only bilinguals were able to learn the new association.

These results reinforce the findings from Study 1. We observed further evidence for monolingual–bilingual differences in establishing and updating a learned behavior, pointing to the importance of the initial learning trajectories during the Training phase. Our fine-grained analytic approach captured additional effects that were undetected in the original analysis, which was performed with trial-level means.

## STUDY 2B: KALASHNIKOVA ET AL. (2020) VISUAL CONDITION

The experimental design of Kalashnikova and colleagues’ (2020, 2021) Visual Condition was very similar to Kovács and Mehler (2009a, Experiment 3). In both studies, infants were cued with a series of three geometric shapes presented silently in an AAB or ABB pattern before a reward appeared consistently on one side of the screen during Training, switching sides at Test. Kalashnikova et al. (2020, 2021) ran 12 trials in each experimental phase and reported block-level analyses. Each block summarized four trials and each phase included three blocks. After applying our participant-level filtering criteria, we retained 41 infants (monolingual = 25, bilingual = 16) from the original sample of 70. We then used our logistic mixed-effects models to analyze the data.

**Figure 5.**
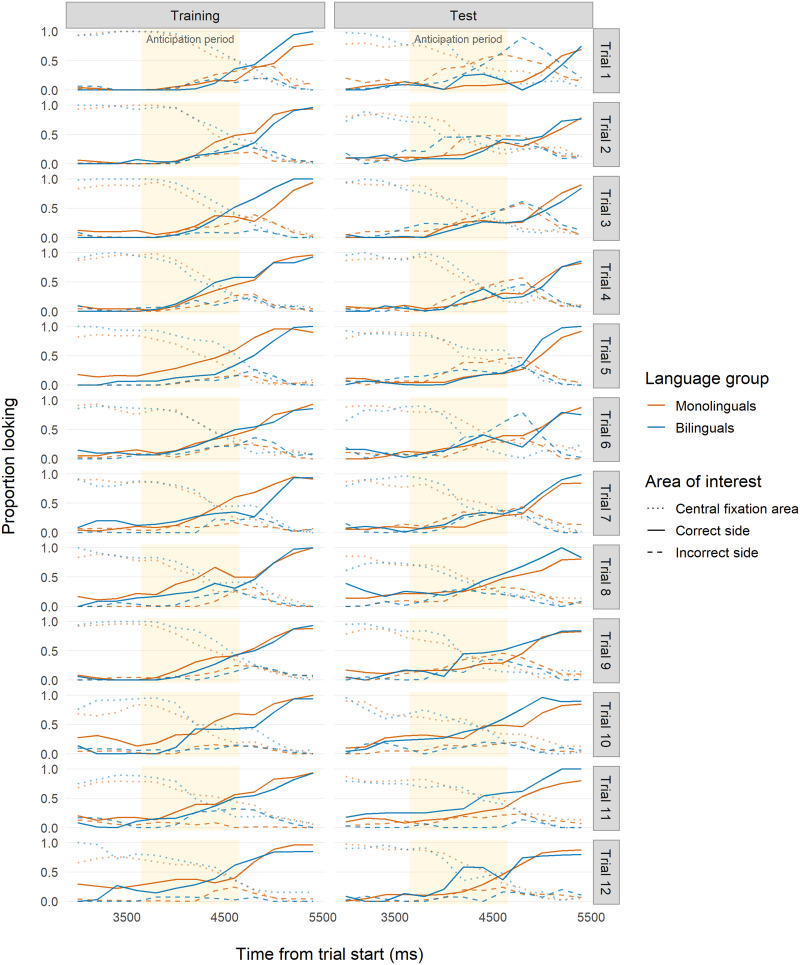
**Time course of infant looking for Study 2b (age 7 months; Visual Condition).** Time course of proportion looking for Study 2b, Visual Condition (reanalysis of Kalashnikova et al., 2020). Red indicates monolingual infants and blue indicates bilingual infants. Solid lines indicate looks to the correct side. Dashed lines indicate looks to the incorrect side. Dotted lines indicate looks to the location where the central fixation cue had appeared. Yellow backgrounds indicate the analyzed anticipation period time frame (3,650–4,650 ms after trial onset) used for our analyses.

During Training (Table 2; Figure 5), monolinguals performed slightly better than bilinguals within trials, an effect not detected by the original block-level analysis. Monolinguals also outperformed bilinguals when measured across trials. However, the difference between monolingual and bilingual performance here was much weaker than the pattern found during Training in Studies 1 and 2a, despite the procedural similarity between studies. At Test (Table 3; Figure 5), bilinguals were faster than monolinguals in updating their previously learned anticipation both within and across trials.

In sum, our reanalysis of Study 2b aligns with the findings from Studies 1 and 2a. Although the effects were less pronounced, they show that monolinguals in this study initially learned to anticipate more quickly than bilinguals, but bilinguals were better at updating their responses when the environment changed.

## STUDY 2C: KALASHNIKOVA ET AL. (2020) AUDITORY CONDITION

In this study, we reanalyzed data from Kalashnikova et al.’s (2020) Auditory Condition. Following Kovács and Mehler’s (2009a, Experiment 1) design, on each trial, infants heard an auditory cue consisting of a different sequence of three syllables in an AAB or ABB pattern that had identical phonetic realizations in Spanish and Basque (the bilinguals’ languages). After applying our filtering criteria for infant attention, we analyzed data from 25 monolinguals and 19 bilinguals.

**Figure 6.**
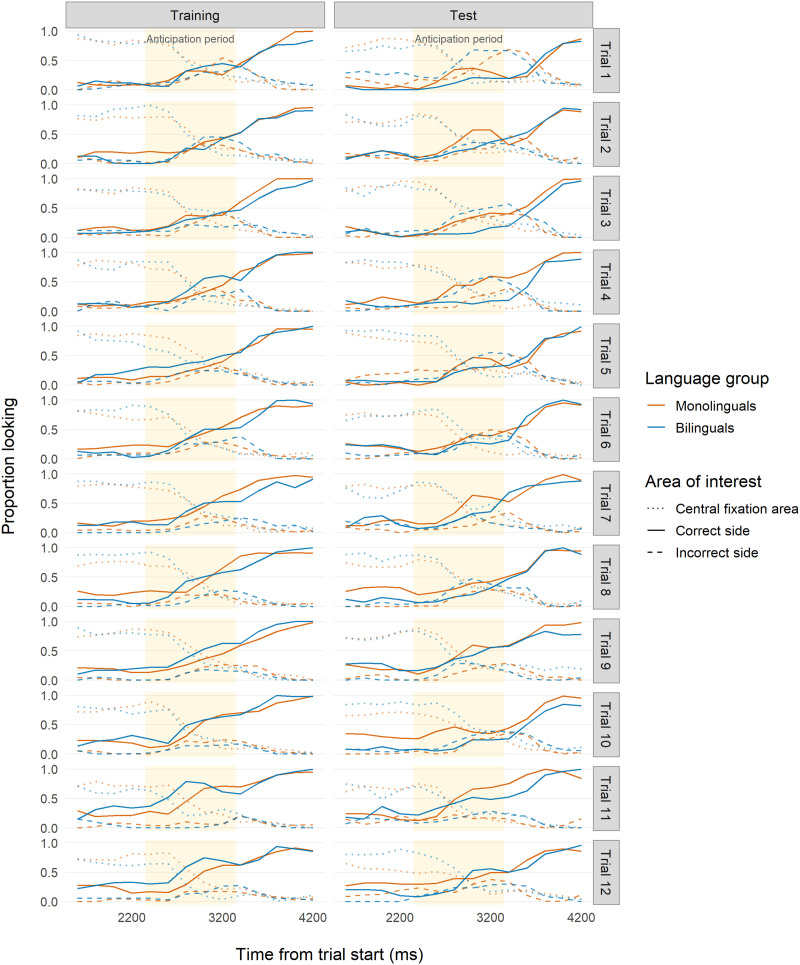
**Time course of infant looking for Study 2c (age 7 months; Auditory Condition).** Time course of proportion looking for Study 2c (reanalysis of Kalashnikova et al., 2020, Auditory Condition). Red indicates monolingual infants and blue indicates bilingual infants. Solid lines indicate looks to the correct side. Dashed lines indicate looks to the incorrect side. Dotted lines indicate looks to the location where the central fixation cue had appeared. Yellow backgrounds indicate the analyzed anticipation period time frame (2,350–3,350 ms after trial onset) used for our analyses.

Using our logistic mixed-effects model approach, we found an opposite trend to the one found in the Training phase of Studies 1, 2a, and 2b (Table 2; Figure 6). Bilinguals outperformed monolinguals within trials and across trials.^2^ However, monolinguals’ odds increased in later time bins of later trials, while bilinguals’ odds decreased slightly. The significant interaction term indicated that by the end of Training, monolinguals’ performance approached bilinguals’.

Also contrary to what was found in Studies 1, 2a, and 2b, but consistent with what was reported by Kalashnikova et al. (2020), monolinguals outperformed bilinguals at Test (Table 3; Figure 6). Monolinguals were substantially faster in updating their previously learned anticipation within trials. Both monolinguals and bilinguals improved across trials, although the effect was slightly stronger for bilinguals. The significant interaction term showed that bilinguals approached monolinguals’ performance by Trial 9. Overall, monolinguals were more successful at updating their previously learned anticipation, although bilinguals did show learning over the course of the Test phase.

In sum, the pattern of results in Kalashnikova et al.’s (2020) Auditory Condition was opposite to the pattern found in the Visual Condition (Study 2b): bilinguals showed faster and more robust learning during Training, but monolinguals were better at updating their response at Test. Together, the results from Studies 1 and 2 highlight a trade-off between performance in the two phases: groups of infants who learn faster in the initial Training phase are not as able to update their anticipation in the subsequent Test phase, and vice versa. This is especially clear in Studies 2b and 2c, which used a within-subjects design. In the general discussion, we elaborate on the common patterns found in Study 1 and 2 (see Table 1 for a summary). In Study 3, we examined developmental effects on the bilingual–monolingual differences we found in Studies 1 and 2, by testing older infants on the same task.

## STUDY 3: 20-MONTH-OLDS, NEW DATA

The goal of this study was to investigate developmental effects on cognition in monolinguals and bilinguals, by testing 20-month-old infants using the same methods as in Study 1. As in the previous studies, we explored the effect of our filtering criteria (reported in the Supplemental Materials and summarized in Table 4). Overall, the pattern of results was weaker with the unfiltered dataset than with the filtered dataset used for the main analyses (i.e., fewer effects reached statistical significance), a point we return to in the discussion.

### Method

#### Participants.

A total of 119 infants were tested. We filtered out 31 infants from the main analysis because they failed to provide sufficient data for both the Training and the Test phases, as defined in Study 1 based on our predetermined criteria. An additional 47 infants were excluded due to fussiness (*n* = 6), health issues (*n* = 2), failure to meet language criteria for being classified as monolingual or bilingual as described in Study 1 (*n* = 35), experimental error (*n* = 1), and parental interference (*n* = 3). All infants were born full-term (at least 37 weeks’ gestation). Data were collected from January 2017 and March 2019. Participants lived in Montreal, Canada, and were recruited from government birth lists, community events, and social media.

The final sample included 41 infants (*M*_age_ = 20m 14d, range: 19m 23d–21m 6d, 21 girls). Twenty-two were monolinguals (*M*_age_ = 20m 15d, range: 19m 24d–21m 6d, 15 girls), exposed at least 90% of the time to either French (*n* = 15) or English (*n* = 7). Nineteen were bilinguals (*M*_age_ = 20m 13, range: 19m 23d–21m 5d, girls = 6), exposed to two languages at least 25% of the time each (English and French, *n* = 16; other language pairs, *n* = 3), hearing both languages from birth (see the Supplemental Materials for language backgrounds). The same criteria were used for monolingualism and bilingualism as in Study 1. Monolingual and bilingual samples both came from mid- to high-socioeconomic-status families. On average, mothers of monolingual infants had 15.5 years of education compared to mothers of bilinguals who had 17.3 years, a statistically significant difference (*t*(39) = −2.87, *p* = .007, *d* = −0.45), although we note that both groups of mothers were highly educated, given that most had completed a bachelor’s or advanced degree (e.g., MA or PhD). Detailed measures are reported in the Supplemental Materials.

#### Measures.

In addition to measuring infants’ language exposure as in Study 1, parents completed a measure of infants’ productive vocabulary, using the MacArthur-Bates Communicative Developmental Inventory (MCDI; Fenson et al., 1993) and/or its adaptation in Québec French (Trudeau et al., 1999), depending on which language(s) their child was learning. Word vocabulary (the sum of unique word forms produced in any language; Gonzalez-Barrero et al., 2020; Hoff et al., 2012) was calculated for monolinguals and French–English bilinguals. Word vocabulary was not calculated for 3 bilinguals learning other language pairs, as the MCDI was only available in one of their languages. Infants with missing vocabulary data were excluded from a preliminary model that tested for effects of vocabulary size (see the Supplemental Materials), but were included in the main model.

#### Stimuli, Apparatus, and Procedure.

The same stimuli, apparatus, and procedure as in Study 1 were used.

### Results

Preliminary analyses indicated no effects or interactions with vocabulary size (details in the Supplemental Materials), so we applied the same analytic strategies as in Study 1.

#### ANOVA.

**Figure 7.**
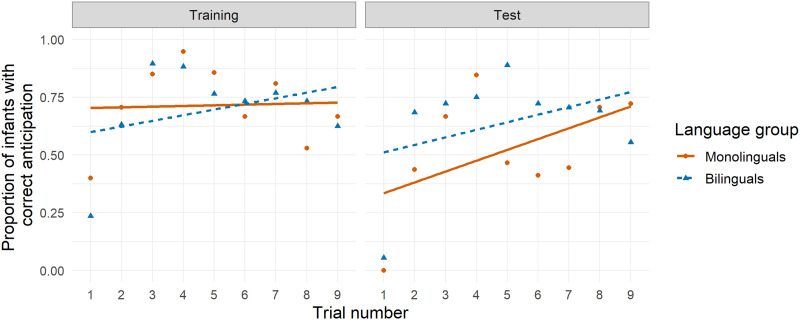
**Correct anticipation by trial for Study 3 (age 20 months).** Visualization of Study 3 (20 months) under Analytic Approach 1 (ANOVAs), similar to Figure 2 from Kovács and Mehler (2009a). Symbols represent the proportion of infants with correct anticipatory looks, with monolinguals plotted in red (circles and solid lies) and bilinguals plotted in blue (triangles and dashed lines). For consistency, lines show best linear fit for each group, although data do not appear to follow a linear trend.

Five participants were excluded from ANOVA analyses due to missing data. During Training (Figure 7), performance increased significantly from Block 1 to Block 2 before falling for Block 3, which could indicate learning over the course of the trials. There were no other significant main effects or interactions. During Test, there were significant main effects of both block and language group, indicating that both language groups learned the association, but that bilinguals’ performance was better overall. Thus, some monolingual–bilingual differences seen at 7 months of age seem to persist at 20 months of age.

#### Logistic Mixed-Effects Regression.

**Figure 8.**
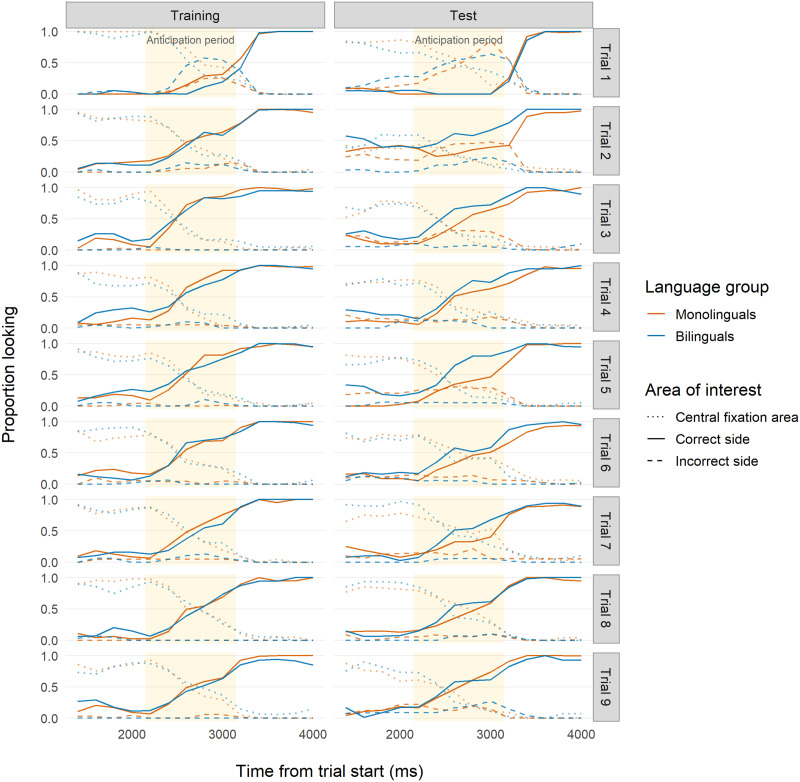
**Time course of infant looking for Study 3 (20 months).** Red indicates monolingual infants and blue indicates bilingual infants. Solid lines indicate looks to the correct side. Dashed lines indicate looks to the incorrect side. Dotted lines indicate looks to the location where the central fixation cue had appeared. Yellow backgrounds indicate the analyzed anticipation period time frame (2,150–3,150 ms after trial onset) used for our analyses.

Overall, 20-month-old infants showed fast learning during Training (Table 2; Figure 8). The model intercept was substantially higher than the intercepts found in Studies 1 and 2, meaning that during the middle time bin of the reference trial (Trial 2), 20-month-old monolinguals made more correct anticipations than 7- to 9-month-olds. Once again, the model odds ratios suggest that monolinguals learned more quickly than bilinguals during Training. Unexpectedly, across trials, the odds of correctly anticipating decreased for both groups, indicating that at 20 months old, infants learned the association quickly and then possibly grew bored of the task. At Test (Table 3; Figure 8), bilinguals performed better than monolinguals on average, and the two groups showed similar improvements within trials. Bilinguals, however, had lower odds of correctly anticipating across trials, again, possibly indicating boredom with the task.

In sum, monolinguals were slightly better at correctly anticipating during the Training phase and bilinguals were slightly faster than monolinguals in updating their previously learned anticipation at Test. This suggests that the effects of bilingualism on cognition observed in younger infants (7- to 9-month-olds) are also observable at an older age (20 months), although they may be less pronounced.

## GENERAL DISCUSSION

This research sought further evidence regarding the effects of bilingualism on infant cognition. To this end, we used an anticipatory eye movement paradigm adapted from Kovács and Mehler (2009a): trials began with a central visual cue, progressed to an anticipatory period, and ended with a visual reward displayed consistently on one side of the screen. At Test, the reward switched to the other side. We collected data from preverbal (7-month-olds; Study 1) and early verbal (20-month-olds; Study 3) bilingual and monolingual infants, which allowed us to examine the impacts of bilingualism at two points in infant development. We also reanalyzed data from three similar studies with 7- to 9-month-old infants for direct comparison of our statistically powerful analytic approach using logistic mixed-effects models (i.e., D’Souza et al., 2020, Experiment 1, which we present in Study 2a; Kalashnikova et al., 2020, Visual Condition, which we present in Study 2b; Kalashnikova et al., 2020, Auditory Condition, which we present in Study 2c).

Confirming our predictions, in four out of the five datasets we analyzed (Studies 1, 2a, 2b, and 3), 7- to 9- and 20-month-old bilinguals were faster and more accurate than monolinguals at updating their anticipatory behavior at Test. Unexpectedly, we found that in three of these same studies, 7- to 9-month-old bilinguals were slower than monolinguals to learn the initial anticipatory response during Training. For 20-month-olds, we saw similar but less pronounced monolingual–bilingual differences during the Training phase. Overall, our results provide compelling evidence that bilingualism affects infant cognitive development, both at younger (7–9 months) and older (20 months) ages. These studies both replicate and extend Kovács and Mehler’s (2009a) study, as we show that this finding is generalizable across methodological variations in the basic paradigm. For example, whereas D’Souza et al. (2020) and Kalashnikova et al.’s (2020) Visual Condition cued infants with a series of static geometric shapes in AAB or ABB patterns presented in silence, in our studies infants were cued with a looming circle paired with a whistle sound. Further, the same pattern of results generalized across diverse bilingual samples, who were learning a variety of language pairs, exposed to a varying prevalence of bilingualism in their communities, and tested in different labs.

Contrary to our predictions, we found the reverse pattern of results in one dataset: Kalashnikova et al.’s (2020) Auditory Condition (Study 2c). In this study, bilinguals outperformed monolinguals during training, but monolinguals outperformed bilinguals at Test. How can we explain this result? Kalashnikova et al. proposed that individual patterns of language exposure—for example, whether infants’ exposure is balanced and whether they come from a bilingual community—could contribute to performance differences in this task. However, if that were the case, we would expect similar performance in the Auditory and Visual Conditions, as the same infants were tested on both conditions in a within-subjects design. Another possible explanation pertains to the nature of the Auditory versus Visual Conditions. Perhaps for linguistic stimuli, which were used in Kalashnikova et al.’s Auditory Condition but not in the other studies analyzed here, bilinguals are faster than monolinguals at initially encoding the information, whereas for nonlinguistic stimuli they are slower (see also Hilchey & Klein, 2011, for a discussion of monolingual–bilingual differences on cognitive tasks with linguistic vs. nonlinguistic stimuli). However, Kalashnikova et al.’s Auditory Condition was a close replication of Kovács and Mehler’s (2009a) Experiment 2, where the opposite, more prevalent pattern was found: bilinguals outperformed monolinguals at Test (although contrary to what we observed, there were no reported differences during Training). While the results of the Auditory Condition raise the possibility that bilingual adaptations to changes in the environment are domain-specific, more research is needed to determine if the pattern of results from this study will replicate with diverse samples and methods.

A consistent finding across Studies 1 and 2 was a trade-off in performance between the Training and Test phases. Groups that showed stronger performance (i.e., faster learning, and perhaps stronger encoding) during Training had more difficulty learning the new association at Test, and vice-versa. In three out of four studies, all with nonlinguistic stimuli, monolinguals were faster than bilinguals to learn the contingency between the cue and the reward during the Training phase. This would have given them more time to strengthen their anticipatory response, which in turn could have led to more difficulty updating their response to a new contingency at test. Inversely, bilinguals might have had weaker initial representations and responses, and thus found it easier to learn a new contingency. The reverse could be true for the one study that used linguistic stimuli (Study 2c). These differences in learning strategies between bilinguals and monolinguals are in line with previous studies that have also reported trade-offs for bilingual children (Struys et al., 2018) and adults (Leivada et al., 2020).

Overall, our results are difficult to explain under theories that view executive functions as the locus of monolingual–bilingual cognitive differences (e.g., Bialystok, 2017). Kovács and Mehler (2009a) interpreted their original results as showing that bilingualism enhances cognitive control even in infancy, arguing that bilinguals were more able than monolinguals to inhibit their original response and learn a new response. However, this interpretation rested on the two groups performing similarly in the Training phase—a finding that we did not replicate. Instead, we found that monolinguals and bilinguals already showed behavioral differences during Training, a simple learning task that does not place any particular demands on inhibition or cognitive control. While these findings do not negate the potential role of executive function in monolingual–bilingual differences, such theories do not provide a complete explanation for the pattern of results we observed.

A compelling hypothesis that is more consistent with our data was advanced by D’Souza et al. (2020): bilingual infants might become more active in sampling multiple sources of information as they interact with a more variable linguistic (and possibly sociocultural) environment. It could be the case that bilinguals required more information during the Training phase before responding, and were thus slower in associating the cue and the reward (at least when nonlinguistic stimuli were used). This experience-dependent adaptive pattern could explain overall differences between monolinguals’ and bilinguals’ anticipations and might impact different areas of cognition (domain-general; Kovács & Mehler, 2009a). Indeed, monolingual–bilingual differences have been reported for a range of abilities, for example, in stimulus encoding and recognition (Singh et al., 2014), memory generalization (Brito & Barr, 2012; Brito et al., 2015; Brito et al., 2020), associative learning (Kovács & Mehler, 2009b), and executive function (Poulin-Dubois et al., 2011). Importantly, the hypothesis proposed by D’Souza et al. shifts the focus from the specific, and hard to define, cognitive ability that the anticipatory looking paradigm measures (e.g., inhibition, cognitive flexibility, attentional processes) to a broader behavioral pattern (exploration) and to environmental variables that might explain it. In this direction, future research could use infant-controlled paradigms to more directly compare how monolinguals and bilinguals explore environments with different levels of complexity (e.g., Kidd et al., 2012).

Our findings have implications for future replications and open science in general. Many recent open science endeavors in infant research (e.g., ManyBabies Consortium, 2020) have focused on reducing false positives (Type I error) that might arise from a number of factors, which is important given the typical small samples in infant research. However, our research provides several directions for increasing statistical power independent of the number of infants tested.

First, we show that some standard analytic approaches may inadvertently increase the chance of false negatives (Type II error; Jaeger, 2008). The nuanced pattern of results from all five studies was only found when using an analytic approach that harnesses the rich moment-to-moment eye-tracking data (up to 80–110 data points per infant; see also Barr, 2008; Humphrey & Swingley, 2018, for related approaches; and Wood, 2017, for a nonlinear approach), which was not revealed in the original trial-level analysis reported by D’Souza et al. (2020, Experiment 1; up to 18 data points per infant) or the block-level analysis reported by Kalashnikova et al. (2020; up to 6 data points per infant).

Second, we were able to examine the effects of applying strict versus loose inclusion criteria (i.e., analyzing data from all infants versus only trials and infants with a minimum level of attention; see Table 4; see also ManyBabies Consortium, 2020, for evidence that infants who contribute more trials show larger effect sizes). For 7- to 9-month-olds, both strict and loose criteria led to similar effect size estimates and patterns of statistical significance in most cases. However, for 20-month-olds, who appeared less engaged in the task, some effects only reached significance when strict inclusion criteria were applied. Applying strict filtering criteria meant higher exclusion rates (reducing sample sizes by 34–48%), but it ensured that analyzed data came from an on-task sample that had enough experience with the task to learn its contingencies. The effects of filtering data in this way likely depend on the study design, the age of the infants, and the analytic technique. It will be helpful for future studies to also systematically compare filtered versus unfiltered data.

Third, we observed striking differences in the datasets of 7- to 9-month-olds we analyzed, both in terms of effect sizes (e.g., the reference, or monolingual, odds of correctly anticipating with time bin ranging from 1.43 to 6.47, with nonoverlapping confidence intervals) and proportions of variance explained across different studies (10–32% of fixed effects; 45–81% of fixed and random effects). This supports what most infant researchers know from experience: seemingly small details of experimental design and/or infant populations can have a large impact on infant performance. Systematic comparisons of different design variables, beyond small pilot studies, will be important in further increasing statistical power in infant research (Bergmann et al., 2018). Furthermore, the high level of measurement error common in infant research, together with small sample sizes, increases the rates of false positives in this field, while also making it more difficult to detect true effects (Byers-Heinlein, Bergmann, & Savalei, 2021). Larger samples or multiple replications using smaller samples (the approach we took here) are crucial for drawing strong conclusions (Bergmann et al., 2018).

Finally, our reanalyses were only possible because other researchers shared their datasets and answered our queries—open science practices that allowed us to make new discoveries (see Bolibaugh et al., 2021, and Dal Ben et al., 2022, for recent discussions of open data and materials in bilingualism research). We hope that future researchers also benefit from the datasets we have generated and shared. In an open science spirit, further large-scale, pre-registered experimentation with infants from a range of ages, cultures, languages, and using standardized stimuli and analytic approaches could also be performed (e.g., Byers-Heinlein et al., 2020; Byers-Heinlein, Tsui, et al., 2021; ManyBabies Consortium, 2020). This approach could help to better understand experimental-level moderators that might affect performance during data collection (e.g., different sets of stimuli, experimental design) and infant-level moderators that might affect learning and cognition over development (e.g., different sociolinguistic contexts).

Bilingual infants experience a complex linguistic environment as they navigate between their languages. Our findings further demonstrate that this experience affects bilinguals’ cognitive abilities as early as age 7 months. In two original and three reanalyzed open datasets, we show that bilingual and monolingual infants might have different learning trajectories. Four out of the five datasets we analyzed support the idea that bilinguals build less rigid initial representations of the world, which in turn are easier to update when circumstances change. On the other hand, monolinguals seem to be faster in building and strengthening initial representations, making it harder to update them when circumstances change. However, in one dataset we observed the reverse pattern, leaving open questions for future research. Thus, our results cannot be easily explained under the traditional notion of enhancements in cognitive control or executive function in bilingual infants. Instead, we believe that these bilingual–monolingual differences are adaptations that arise from dealing with environments with different degrees of linguistic (and probably social) variability, and as such can be viewed as either a help or a hindrance, depending on the context. The origins of such adaptations, moderators, and their impact later in life are interesting open questions. Large-scale data collection from diverse bilingual infants, together with analyses that tap into the dynamics of infant learning, have great potential to contribute to the complex and exciting debate of bilingualism’s effects in infancy.

## ACKNOWLEDGMENTS

We thank Dean D’Souza and Marina Kalashnikova for their responsiveness in providing additional demographic information for the reanalyses conducted in Study 2 (a, b, and c). We also thank the members of our lab for their assistance with data collection and feedback on previous versions of the manuscript.

## FUNDING INFORMATION

KBH, Natural Sciences and Engineering Research Council of Canada, Award ID: 402470-2011. KBH, Natural Sciences and Engineering Research Council of Canada, Award ID: 2018-04390. SPI, Fonds de Recherche du Québec-Société et Culture (https://dx.doi.org/10.13039/100008240). SPI, Concordia University (https://dx.doi.org/10.13039/501100002914). RDB, Concordia University (https://dx.doi.org/10.13039/501100002914).

## AUTHOR CONTRIBUTIONS

RDB and HK share first authorship and are listed alphabetically. RDB: Conceptualization: Equal; Data curation: Equal; Formal analysis: Equal; Software: Equal; Validation: Equal; Writing – original draft: Equal; Writing – review & editing: Equal. HK: Data curation: Lead; Formal analysis: Lead; Software: Lead; Validation: Equal; Visualization: Lead; Writing – original draft: Equal; Writing – review & editing: Equal. SPI: Conceptualization: Lead; Investigation: Lead; Methodology: Equal. KBH: Conceptualization: Lead; Funding acquisition: Lead; Project administration: Lead; Resources: Lead; Supervision: Lead; Writing – original draft: Equal; Writing – review & editing: Equal.

## Notes

^1^ Weighted proportion looking to the correct side was the dependent variable. The proportion of looking to the correct side (ranging from 0 to 1) was calculated for each time bin by averaging the values of all samples (binary, either 0 or 1) in a given bin. This resulted in a quasi-binomial distribution and the number of samples was used to weight the proportions between 0 and 1 (see analysis scripts at https://osf.io/bz8jn).^2^ We experienced convergence issues with the Study 2c Training phase model. However, the model converged when the trial number variable was rescaled (trial number/2), resulting in very similar direction and magnitude for all estimates. To be consistent, here we report the unscaled model, and interested readers can see the scaled model results in the analysis code scripts.

## Supplemental Materials

https://osf.io/bz8jn
